# MYCN: from oncoprotein to tumor-associated antigen

**DOI:** 10.3389/fonc.2012.00174

**Published:** 2012-11-16

**Authors:** Vito Pistoia, Fabio Morandi, Annalisa Pezzolo, Lizzia Raffaghello, Ignazia Prigione

**Affiliations:** Laboratory of Oncology, Translational Research and Laboratory Medicine, G. Gaslini InstituteGenoa, Italy

**Keywords:** MYCN oncoprotein, neuroblastoma, tumor antigen, cytotoxic T cells, immunotherapy

## Abstract

MYCN is a well-known oncogene over-expressed in different human malignancies including neuroblastoma (NB), rhabdomyosarcoma, medulloblastoma, astrocytoma, Wilms’ tumor, and small cell lung cancer. In the case of NB, MYCN amplification is an established biomarker of poor-prognosis. MYCN belongs to a family of transcription factors (the most important of which is C-MYC) that show a high degree of homology. Down-regulation of MYC protein expression leads to tumor regression in animal models, indicating that MYC proteins represent interesting therapeutic targets. Pre-requisites for a candidate tumor-associated antigen (TAA) to be targeted by immunotherapeutic approaches are the following, (i) expression should be tumor-restricted, (ii) the putative TAA should be up-regulated in cancer cells, and (iii) protein should be processed into immunogenic peptides capable of associating to major histocompatibility complex molecules with high affinity. Indeed, the MYCN protein is not expressed in human adult tissues and up-regulated variably in NB cells, and MYCN peptides capable of associating to HLA-A1 or HLA-A2 molecules with high affinity have been identified. Thus the MYCN protein qualifies as putative TAA in NB. Additional issues that determine the feasibility of targeting a putative TAA with cytotoxic T lymphocytes (CTLs) and will be here discussed are the following, (i) the inadequacy of tumor cells *per se* to act as antigen-presenting cells witnessed, in the case of NB cells, by the low to absent expression of HLA class I molecules, the lack of co-stimulatory molecules and multiple defects in the HLA class I related antigen processing machinery, and (ii) the immune evasion mechanisms operated by cancer cells to fool the host immune system, such as up-regulation of soluble immunosuppressive molecules (e.g., soluble MICA and HLA-G in the case of NB) or generation of immunosuppressive cells in the tumor microenvironment. A final issue that deserves consideration is the strategy used to generate CTL.

## INTRODUCTION

*MYCN* is a well-known oncogene over-expressed in different human malignancies including neuroblastoma (NB), rhabdomyosarcoma, medulloblastoma, Wilms’ tumor, and small cell lung cancer (Lee et al., 1984; Garson et al., 1985; Nisen et al., 1986; Wong et al., 1986; Williamson et al., 2005). In the case of NB, *MYCN* amplification is an established prognostic biomarker that helps stratify patients in different risk categories (Brodeur et al., 1984).

MYCN belongs to a family of transcription factors (the most important of which is C-MYC) with high degree of structural homology (Malynn et al., 2000). Down-regulation of MYC protein expression in different animal models of human malignancies leads to tumor regression, indicating that MYC proteins represent interesting therapeutic targets (Pelengaris et al., 1999; Morgenstern and Anderson, 2006).

Transgenic expression of the human *MYCN* gene in mice under the control of the tyrosine hydroxylase (TH) promoter is followed by the spontaneous development of *MYCN*-amplified human NB in the adrenal gland, that mimics that natural history of primary NB (Weiss et al., 1997). This innovative model has allowed to broaden knowledge on NB pathogenesis and design therapeutic strategies aimed at inhibiting expression of MYCN protein and consequently dampening tumor growth. Such strategies are discussed in detail in other articles of this issue of Frontiers in Oncology and are beyond the scope of this review.

The immune system has evolved into two major arms making use of innate and adaptive mechanisms, respectively, in order to cope with pathogens. The adaptive immune system is composed of B and T lymphocytes that interact specifically with antigens through two different sets of antigen receptors, i.e., the B cell receptor (BCR) and the T cell receptor (TCR), respectively (Abbas et al., 2011). At variance with the BCR that recognizes native protein antigens, the TCR binds to small peptides generated following antigen processing by antigen-presenting cells (APCs). These peptides associate with nascent major histocompatibility complex (MHC) molecules and are presented to the TCR on T cells (Abbas et al., 2011). Antigen-derived peptides must fit in size within small pockets present in T cell-associated MHC class I or II molecules. Peptide binding to TCR (first signal) causes T cell activation only when co-stimulatory molecules present on APCs are engaged by their ligands on T cells (second signal). In the absence of the latter interactions, T cells that have bound antigen-derived peptides undergo apoptosis or anergy (Abbas et al., 2011).

Tumor growth is assisted by different immune evasion mechanisms that frustrate the attempts of the host immune system to eliminate cancer cells (Schreiber et al., 2011). Major goals of tumor immunology are to unravel such mechanisms and to develop immunotherapeutic approaches leading to tumor regression.

Based upon this background, we discuss here MYCN as potential target for NB immunotherapy.

## THE MYCN PROTEIN AS PUTATIVE TUMOR-ASSOCIATED ANTIGEN

Malignant cells express antigens shared with their normal counterparts and antigens that are tumor-restricted since their expression is reactivated in cancer cells only, e.g., embryonal antigens. In order to hit selectively neoplastic cells, immunotherapy must target tumor-associated antigens (TAAs) present almost exclusively on tumor cells. A good example in this respect is represented by anti-GD2 monoclonal antibodies used for therapy of high risk NB patients. GD2 is a ganglioside expressed in the central nervous system that shows a very limited expression in the peripheral tissues and is therefore amenable to be targeted for NB treatment (Seeger, 2011). Anti-TAA monoclonal antibodies kill tumor cells by promoting antibody-dependent cell cytotoxicity, operated predominantly by NK cells and macrophages that bind the Fc portion of cancer cell-coating IgG through specific surface receptors. An additional mechanism involved in the anti-tumor activity of anti-TAA antibodies is complement-dependent cell cytotoxicity.

A second pre-requisite for a candidate TAA is its expression at high level on cancer cells which reinforces interaction between BCR or TCR and the putative TAA.

Finally, T cell-targeted TAA must be processed into immunogenic peptides capable of associating with high affinity to MHC molecules, thus allowing strong binding of the MHC–peptide complex to TCR. Selection of the best peptides for T cell-based immunotherapy is facilitated by bioinformatics tools that predict the peptide affinity for MHC molecules.

During murine development, MYCN is expressed in different tissues such as the heart, the neural tube, and the limb buds. At birth, MYCN expression is detected in brain, intestine, kidney, heart, and lung, but is lost in the first weeks of life (Jakobovits et al., 1985; Zimmerman et al., 1986, 1990; Semsei et al., 1989). Recently, Anderson and colleagues have provided further evidence that MYCN mRNA is virtually undetectable in most adult human tissues, at variance with rhabdomyosarcoma and NB cell lines that over-express this oncogene (Himoudi et al., 2008). Thus, MYCN behaves as an embryonal TAA whose expression is down-regulated shortly after birth and up-regulated in cancer cells of neuroectodermal origin, including NB, through different mechanisms such as gene amplification.

Two MYCN-derived peptides capable of eliciting cytotoxic T lymphocyte (CTL) responses against NB cells have been identified (Sarkar and Nuchtern, 2000; Himoudi et al., 2008). Nuchtern and colleagues selected on the ground of its long-life and synthesized the nine amino acid MYCN-derived S69K peptide that contains an HLA-A1 binding motif. S69K was then used to generate CTL from peripheral blood mononuclear cells of an HLA-A1^+^ normal donor and an HLA-A1^+^ NB patient carrying a *MYCN*-amplified tumor (Sarkar and Nuchtern, 2000). Recognition of tumor cells by these CTL was found to depend on *MYCN* amplification and HLA specificity, since CTL lysed only *MYCN*-amplified primary NB cells that expressed HLA-A1. The immunophenotype of S69K peptide-stimulated CTL was found to be CD3^+^, CD8^+^. Since NB cells display very low expression of HLA class I molecules (see below), Nuchtern and colleagues investigated whether incubation of tumor cells with interferon-γ (IFN-γ), that up-regulates HLA class I expression, improved the rate of lysis operated by S69K peptide-specific CTL. These experiments showed that IFN-γ pre-treated NB cells were killed twice more efficiently than untreated NB cells, but pre-incubation of tumor cells with the cytokine was dispensable for lysis to occur. Thus, low level expression of HLA class I on NB cells does not preclude recognition of at least a subset of them by S69K peptide-specific CTL (Sarkar and Nuchtern, 2000).

Using a bioinformatics approach, Anderson and colleagues identified in the MYCN sequence the 10 amino acid VIL peptide, predicted to bind HLA-A2 (Himoudi et al., 2008). This peptide showed favorable binding activity and the VIL epitope was not detected in any other human protein sequence including C-MYC. VIL-specific CTL were generated from two normal donors and a child with advanced *MYCN*-amplified NB using the following protocol. First autologous dendritic cells (DCs) and then autologous B cells and irradiated feeder cells were pulsed with the peptide in the presence of IL-2 and IL-7 for the first 2 weeks of culture. Peptide pulsing was repeated in the second 2 weeks using a different cytokine *milieu*, i.e., IL-2 and IL-12. Anderson and colleagues showed that VIL-specific CTL lysed specifically *MYCN*-amplified target cells in an HLA-A2-restricted manner. Furthermore, VIL-specific CTL released IFN-γ upon incubation with the same target cells, suggesting that this cytokine might improve the efficiency of NB cell killing by up-regulating HLA class I expression on NB cells (Himoudi et al., 2008).

Taken together, these results indicate that (i) MYCN is a good target for T cell mediated anti-NB immunotherapy, although this potential approach is limited to patients carrying *MYCN*-amplified tumors, (ii) the HLA-A1 and HLA-A2-restricted MYCN-derived peptides identified by the groups of Nuchtern and Anderson, respectively, can be used to treat more than half Caucasian patients due to the widespread distribution of these alleles in the general population, and (iii) HLA class I expression on NB cells is low, but this does not preclude lysis of a subpopulation of *MYCN*-amplified NB cells by MYCN peptide-specific CTL.

## IMMUNE EVASION MECHANISMS OPERATED BY NEUROBLASTOMA TUMORS

As anticipated, tumor growth is facilitated by cancer cell-driven immunosuppressive mechanisms. In the tumor microenvironment, interactions between malignant cells and stromal cells mediated by soluble factors and direct cell to cell contact modulate macrophages, DCs, NK cells, cytotoxic, and helper T lymphocytes toward a tolerogenic phenotype while expanding immunosuppressive cell populations such as T regulatory cells and myeloid-derived suppressor cells (Schreiber et al., 2011). In this respect, it has been shown that cytokines as IL-6, IL-10, and TGFβ1 are over-expressed in poor-prognosis NB cases, and that IL-6 promotes NB growth and metastasis through different mechanisms (Seeger, 2011).

In the last 10 years we have contributed to elucidate the immune evasion mechanisms adopted by NB cells to escape the control of the host immune system. These studies have highlighted (i) the release of soluble molecules suppressing anti-tumor immune reactivity, such as the stress inducible NKG2D ligand MICA (Raffaghello et al., 2004) and the HLA class Ib molecule HLA-G (Morandi et al., 2007) and (ii) NB cell defects in the expression of a few components of the HLA class I related antigen processing machinery (APM; Raffaghello et al., 2005). We will focus on the latter issue since it is closely related to the topic of this article.

Tumor-associated antigens are endogenous proteins processed and presented to CD8^+^ T cells in the context of MHC class I (MHC I) antigens (HLA class I in humans). In brief, MHC I peptide generation starts with polypeptide degradation by the proteasome in the cytosol. Peptides generated by proteasomal digestion subsequently undergo structural modifications operated by different peptidases and bind to the transporter of antigen processing (TAP), a heterodimer composed of two different subunits named TAP1 and TAP2. TAP-bound peptides are translocated into the endoplasmic reticulum or the Golgi where they are further trimmed by aminopeptidases. Peptides of appropriate length (8–11 mers) that are able to bind MHC I allelic products expressed by the cell are inserted into MHC I proteins, formed in humans by the association of HLA class I heavy chain (HC) and β2-microglobulin (β2m), and are exported to the cell surface. Peptide insertion in MHC I molecules is driven by a loading complex that includes different chaperons such as tapasin, calnexin, calreticulin, and Erp-57 (Tanaka and Kasahara, 1998; De Verteuil et al., 2012).

All eukaryotic cells possess constitutive proteasomes with a 20S hollow proteolytic core providing a cavity open at both ends in which proteins are degraded. The 20S proteasome is composed of 14 different subunits organized in a barrel-shaped complex with the stoichiometry α7β7β7α7. Three subunits of the inner ring, i.e., β1, β2, and β5 are directly involved in proteolytic cleavage. In cells exposed to IFN-γ, these latter subunits are replaced by LMP2 for β1, MECL1 (or LMP10) for β2 and LMP7 for β5 to give rise to the 26S immunoproteasome, which generates more immunogenic peptides from digested polypeptides. It is of note that constitutive expression of the immunoproteasome is detected in some cell types, such as for example DCs, that represent the most potent professional APCs. Proteasomal subunits, both constitutive and IFN-γ induced, the TAP complex and the chaperons that assist in peptide loading onto MHC I are collectively referred to APM (Tanaka and Kasahara, 1998; De Verteuil et al., 2012).

We investigated APM expression in human NB cells from both primary tumors and continuous cell lines since APM abnormalities, that have been identified in different tumor types, cause defects in peptide generation, translocation, and loading onto β2m–HLA HC complexes. Therefore, these complexes are retained in the endoplasmic reticulum, are degraded by housekeeping proteasomes to a larger extent and are unstable. IFN-γ treatment of tumor cells usually restores peptide supply to β2m-HLA HC complexes by up-regulating expression of APM components and reverses HLA class I down-regulation, that is particularly evident in NB cells (Seliger et al., 2000).

Immunohistochemical staining of a series of stroma-poor NB tumors showed that LMP7, TAP2, β2m, and β2m-free HC, as well as HLA class I and II molecules, were never expressed in tumor cells. Pediatric adrenal medulla, tested as normal counterpart of NB cells, expressed all these APM components at variable intensity, but tested negative for HLA class I and II. These results indicated that absence of tapasin, TAP2, β2m, and β2m-free HC in primary NB lesions was related to malignant transformation (Raffaghello et al., 2005).

Flow cytometric analysis of a panel of NB cell lines showed that the β1 constitutive proteasomal subunit, LMP2, LMP7, LMP10, TAP1, and Erp-57 were virtually undetectable under basal conditions (Raffaghello et al., 2005). Differences in APM component expression detected in primary NB cells vs NB cell lines (such as for example LMP10 expression in the latter but not the former cells, and Erp57 expression in the former but not the latter cells) may be related to the different sensitivity of immunohistochemistry vs flow cytometry and/or to changes acquired by primary NB cells during their transformation in immortal cell lines or later on due to adaption to culture conditions.

Incubation of NB cell lines with IFN-γ caused up-regulation of LMP2, tapasin, β2m, and β2m-free HC, as well as of surface HLA class I. Functional experiments showed that lysis of IFN-γ treated HLA class I negative NB cell lines by NK cells matched for HLA class ligands of killer inhibitory receptors (KIR) was significantly reduced. In contrast, such reduction was not observed when the same cell lines that had been pre-treated with IFN-γ were incubated with activated NK cells from healthy donors mismatched for KIR HLA class I ligands (Raffaghello et al., 2005).

In essence, these experiments demonstrated that (i) NB cells display different defects in APM component expression and in surface HLA class I expression that may contribute to altered peptide generation and loading onto HLA class I molecules, (ii) incubation of NB cells with IFN-γ up-regulates the expression of selected APM components and of surface HLA class I molecules. This latter finding may represent a double-edge sword since on one hand IFN-γ induced changes in NB cells that can improve their recognition and lysis by TAA-specific CTL, but on the other hand can reduce sensitivity of tumor cells to NK cell mediated lysis. Such considerations must be kept in mind when designing immunotherapeutic strategies aimed at promoting elimination of NB cells through cytotoxic effector mechanisms operated by the host immune system.

## *MYCN* IS AMPLIFIED NOT ONLY *IN BONA FIDE* NB CELLS BUT ALSO IN NB CELLS DISGUISED AS ENDOTHELIAL CELLS

Previous studies have demonstrated that tumor cells can line channels mimicking endothelial vessels. This phenomenon has been denominated “vascular mimicry” and reported in various tumor types including melanoma, renal carcinoma, B cell non-Hodgkin lymphomas, and fibrosarcoma (Hendrix et al., 2003).

We have recently investigated vascular mimicry in NB and the underlying mechanisms. To detect tumor-derived endothelial cells (TECs) in NB microenvironment we have used a combination of fluorescent *in situ* hybridization (FISH) for *MYCN* and immunofluorescence for endothelial cell marker, mainly CD31 (Pezzolo et al., 2007, 2011).

**FIGURE 1:**
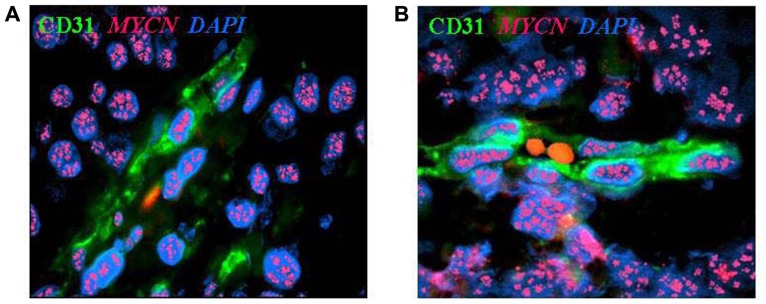
**Neuroblastoma-derived endothelial micro-vessels. (A)** Immunofluorescence and fluorescent *in situ* hybridization analysis of NB tumor section highlights CD31^+^ endothelial micro-vessel (green) carrying *MYCN* amplification (multiple red signals). **(B)** Two RBCs are in the open lumen of the NB-derived endothelial micro-vessel. Nuclei are stained with DAPI (blue), ×100.

Using this approach, we have identified CD31^+^ cells carrying *MYCN* amplification in one-third of *MYCN*-amplified primary NB tested and in tumors formed by *MYCN*-amplified human NB cell lines in immunodeficient mice (**Figures 1A,B**). These TEC lined functional endothelial micro-vessels containing red blood cells in their lumen (**Figure 1B**) and coated by α-smooth muscle actin (SMA)^+^ pericytes that never displayed *MYCN* amplification and were therefore of host origin (**Figure 1**; Pezzolo et al., 2007). An interesting feature of TEC was the co-expression of mature endothelial cell markers [CD31, CD105, von Willebrand factor, prostate-specific membrane antigen (PSMA), HLA class I] together with neuroblastic markers such as GD2, CD56, CD57, and NB84 (Pezzolo et al., 2011).

In these studies, we have identified a small subpopulation of NB cells with perivascular location that express the Oct-4 transcription factor, together with the extracellular matrix protein tenascin C (TNC) on the cell surface. *In vitro* and *in vivo* experiments demonstrated that these Oct-4^+^, TNC^+^ NB cells were endowed with plasticity since they (i) co-expressed “stem cell” markers as SOX-2 and Wnt and formed neurospheres in culture under appropriate conditions, (ii) differentiated *in vitro* into endothelial-like cells expressing vascular endothelial (VE)-cadherin and, in part, CD31 following 2-week culture in the presence of VE growth factor (VEGF) and basic fibroblast growth factor (bFGF), and (iii) formed tumors *in vivo* that contained *MYCN*-amplified TEC, whereas Oct-4^-^, TNC^-^ NB cells gave rise to tumors devoid of TEC (Pezzolo et al., 2011).

All of the above led to the conclusion that Oct-4^+^, TNC^+^ NB cells were progenitor cells that served as reservoir of TEC. The implications of the existence of *MYCN*-amplified TEC are that these cancer cells disguised as endothelial cells are genetically unstable and involved in chemoresistance and tumor progression. Although in our studies we investigated *MYCN* amplification but not MYCN protein expression, it is well-known that *MYCN*-amplified tumor usually over-express the corresponding protein. Thus, targeting of these TEC with MYCN directed immunotherapy may provide an additional benefit to the patient.

## PRACTICAL APPROACHES TO MYCN-TARGETED T CELL IMMUNOTHERAPY

A final issue that must be discussed is how MYCN-targeted T cell immunotherapy can be accomplished and what approach has the best chances of being successful.

MYCN-specific T cells can be generated *ex vivo* starting from patient peripheral blood mononuclear cells, as exemplified by the experiments from the Nuchtern’s and Anderson’s groups, expanded and reinfused into the patient (adoptive immunotherapy; Sarkar and Nuchtern, 2000; Himoudi et al., 2008). Alternatively, the patient immune system can be stimulated to generate MYCN-specific endogenous CD8^+^ T cells.

Myeloid DCs can be generated by culture of patient peripheral blood monocytes in the presence of cytokines as GM-CSF, IL-4, TNF, or IFN-α, pulsed with MYCN peptide and incubated with patient T cells in order to expand MYCN-specific CTL to be reinfused in the patient. This is one of the most popular approaches to cancer immunotherapy due the potent APC function of DCs (Jain, 2010).

A second approach to adoptive immunotherapy is transfection of patient DC with adeno-associated viral vectors, lentiviral vectors or tumor mRNA in order to induce expression of TAA in these APC and promote generation of TAA-specific CTL (Jain, 2010). A few years ago, we conducted a study in which DC generated from monocytes of NB patients or age-matched healthy donors were transfected with mRNA from four pooled NB cell lines and co-cultured with autologous CD8^+^ T lymphocytes (Morandi et al., 2006). Expanded CTL produced IFN-γ upon incubation with HLA-matched NB cell lines and lysed in HLA-restricted manner the same cell line, but not autologous T cell blasts. CTL from normal subjects recognized only peptides from anaplastic lymphoma-associated kinase (ALK) and preferentially expressed antigen of melanoma (PRAME). CTL from NB patients reacted not only to ALK and PRAME peptides but also to peptides from survivin, telomerase, and TH (Morandi et al., 2006). Reactivity of patient or normal CTL to MYCN peptide was not investigated in this study, but it is conceivable that MYCN peptide-specific CTL can be generated using tumor mRNA DC transfection from NB patients carrying *MYCN*-amplified tumors.

The third and most promising approach of adoptive immunotherapy of cancer rests upon the administration to patients of chimeric antigen receptor (CAR)-engineered lymphocytes. CARs usually combine the antigen binding of a monoclonal antibody with the signaling machinery of a T cell. Further modification of last generation CAR include the addition of stimulatory domains that increase activation of CAR-bearing T cells. Gene transfer procedures such as transduction with retrovirus and transfection with plasmids are used to graft CAR into T cells. An advantage of CAR-engineered T lymphocytes is that they kill tumor cells expressing target antigens on the cell surface in a HLA-independent manner. Survival of CAR-engineered T cells after adoptive transfer is improved by lymphodepletion before and provision of T cell growth factor after infusion (Ramos and Dotti, 2011).

Chimeric antigen receptor-engineered T cells expressing a chimeric anti-GD2 antibody on the cell surface have been generated and administered to NB patients in the frame of a phase I study, in which EBV-specific T cells were grafted with the chimeric construct and periodically restimulated with the specific antigen in order to promote their long-term survival. Complete remission was achieved in 3/11 patients with active disease and persistence *in vivo* of GD2 carrying CAR beyond 6 weeks was found to be associated with better clinical response (Pule et al., 2008; Louis et al., 2011).

In principle, this very same strategy can be applied to generate TAA-specific in general, and MYCN peptide-specific in particular, CD8^+^ T cells that will be subsequently grafted with the chimeric anti-GD2 antibody and infused in NB patients carrying *MYCN*-amplified tumors.

Stimulation of the patient immune system to generate endogenous MYCN-specific CTL may be achieved in two ways, (i) administration of TAA peptide and (ii) vaccination with plasmid carrying DNA sequences encoding the target TAA.

Peptide vaccination in cancer cells is feasible and displays a favorable toxicity profile, but patient immunization is achieved only in a half of them. Different approaches have been recently developed to induce stronger peptide-driven immune-mediated control of tumor growth, with special emphasis on multi-peptide-based cancer vaccines. This latter approach presents numerous advantages, such as (i) the possibility of overcoming tumor heterogeneity and selection of antigen-negative clones that have escaped immune responses directed to a single peptide, and (ii) the combination of HLA class I and class II restricted epitopes, thus triggering CD4^+^- and CD8^+^-mediated recognition of cancer cells (Jain, 2010; Perez et al., 2010).

Lode and colleagues have presented an abstract at the Advance in Neuroblastoma Research 2012 meeting reporting on the development of a MYCN-DNA (pMDV1) vaccine based on epitopes encoding for three peptides from the MYCN protein sequence with high affinity to MHC I. This vaccine was tested in a novel syngeneic MYCN over-expressing NXS2 mouse model. MYCN-DNA vaccination was performed before challenge with tumor cells and resulted in reduction of primary tumor growth as well as in antigen-specific target cell killing (Stermann et al., 2012). These preliminary findings may provide a platform for further clinical development.

## CONCLUSION

This review article provides evidence for MYCN protein as potential immunotherapeutic target in NB along different experimental strategies that have been discussed in detail. The time of testing such strategies in the clinical setting has come of age.

## Conflict of Interest Statement

The authors declare that the research was conducted in the absence of any commercial or financial relationships that could be construed as a potential conflict of interest.
